# Acute Toxic Anæmia*Case reported at meeting of Pennsylvania State Veterinary Medical Association, March 7th.

**Published:** 1893-03

**Authors:** Jacob Helmer

**Affiliations:** 311 Spruce Street, Scranton, Pa.


### ACUTE TOXIC ANÆMIA.*

### Was it caused by the *Coleosporium Solidaginis, Thumen.* (Scheinmitz.)

By Dr. Jacob Helmer.

In the month of September, 1891, we received a call from Messrs. Simpson and Clelland to go to their stock farm situated at *Lake Ariel*, in Wayne County, Pa. My brother, R. C. Helmer, received the call and hastily took the last train that day (Saturday) to the lake. On his return that evening he called my attention to a very interesting case.

The patient was a very valuable brood mare, *Lady "MacGregor,"* age seven years, fifteen hands high, and bay in color.

The case was found standing in a loose box ; she was eating some grass that had just been given her.

The first thing noticed was her emaciated condition. The animal was reduced to skin and bones. On examination the pulse was found to be very frequent and weak. The impulse of the heart walls beating upon comparatively empty cavities could be heard a few feet away.

The respirations were increased, temperature slightly sub-normal, visbile mucous membranes pale, almost white, extremities cold. On motion (from the muscular debility) the animal reeled and appeared as if she would fall. There was no pain, no uneasiness. But there was thirst and a ravenous appetite. The kidneys and bowels both performed their functions normally.

On inquiry into the history of the case, it was found that the animal, with others, had been in the pasture field all Summer.

---

* Case reported at meeting of Pennsylvania State Veterinary Medical Association, March 7th.

Sometimes the animals would not be closely inspected for days at a time. This was because they could be seen at a distance from the barns. But on a visit to the animals in that field it was at once noticed that two of the mares were considerably emaciated, especially the one mentioned, "Lady MacGregor." These two were immediately removed to the barn. Five days before this these animals appeared in good health. Three others in the same field were apparently not affected, and one other horse that had just been turned into the field was not affected.

This was the history. In the case of Lady MacGregor it was apparent that she would not live long. She died the following day, *Sunday*. On Monday morning I went to see the cases and made a post-mortem.

*The morbid anatomy* was as follows: Body emaciated, voluntary muscles, pale color, all the internal organs very pallid, the serous surfaces dull and wrinkled, heart diminished in size, pericardial fluid diminished, fatty tissue diminished, cavities almost empty, muscle substance of a pale color, and flabby feel.

The lungs were crepitant and pale, at the base a slight odæma. Liver normal in size, but pale. The gastro-intestinal tract was pale, but studded here and there with ecchymotic spots. The spleen was apparently normal, in size and slightly friable. The lymph glands seemed normal but were not well examined. The brain substance presented a very anæmic appearance. But nothing further was noted. The kidneys presented nothing special. The genital organs the same. The large blood vessels were normal in appearance, but, like the heart, almost empty. The want of blood in the system was a prominent characteristic of the post examination.

It now devolved upon me to give an opinion of the cause of the animal's death. The symptoms produced and the animal's death was decided as probably being caused by a poison that was being taken into the system either through the water or food supply.

As animals in adjoining fields, that drank the same water, were not affected, the idea of the trouble being possibly caused by the water was rejected. But if it were in the food, then the poison must flourish in the field, as that was the only source of food to these animals. One thing was noted. The pasture ranged alongside an undergrowth of woods, into which here and there the animals entered for shade.

The woods, therefore, were first thoroughly inspected, but nothing suspicious was found; nothing there seemed to have been eaten. Even the branches of the bushes were scarcely touched.

Next we turned attention to the other animals in the field. As before stated one of these had already been taken to the barn, but was not in an advanced stage like Lady MacGregor.

The animals still in the field (excepting one which had just been turned in) were affected. The symptoms varied merely in degree. But in each case the pulse was frequent and weak, visible mucous membranes pallid and body emaciated.

In coming to the part of the field where these animals were standing, an abundance of the low variety of the golden rod, *solidago odora*, was all along noticed. My comrades informed me that it was very common throughout the entire field. It was subsequently found to exist liberally on all that side of the farm, an extensive tract. Also upon other farms in that section of the country.

But a peculiarity was noticed in connection with this common plant. It was loaded with a reddish rust. This rust I had often observed, but never before the quantity.

I also knew that horses are not fond of the golden rod, but could find in the fields nothing else to suspicion. However, it was thought best to offer these cases some, and thus determine whether they would eat it or not. Having selected some specimens that were laden with the rust and devoid of admixture with grasses the collection was offered.

The affected animals eagerly devoured it. I had some choice specimens in my coat pockets. These were betrayed by the peculiar odor and were nosed after just as eagerly as if it had been sugar or apples.

The next thing was to offer some to the one animal that was not affected. This gentle fellow would not accept it, but laid back his ears and wheeled on me. However, on approaching him again he accepted grasses from my hand, but not a particle of the weed. *Here, then, was an interesting and noteworthy fact.*

Specimens were next offered the patient in the barn ; these were at once devoured and desire manifested for more. Unaffected animals (on other portions of the farm, but where the rust weed existed only in limited quantity) could not be induced to take a mouthful.

The affected animals were now ordered to the barn. The unaffected one was sent to another field.

The patient taken out of the field at the same time with Lady MacGregor was now, for the first time, examined by me.

It will not be necessary here to repeat the symptoms presented, as they were the same presented by the MacGregor mare and already described. But they differed in degree. They were not so severe, but yet severe enough to warrant an unfavorable prognosis.

The hygienic treatment adopted was plenty of fresh air, also good food and plenty of it. In the way of medicines, tonics were administered, also peroxide of hydrogen subcutaneously.

The other cases were merely given good food and tonics.

One peculiar symptom, in addition to those previously mentioned, was an occasional sinking spell on the part of the animal most affected.

These patients all recovered in time, but it was many months before the symptoms wholly disappeared.

Now we come to a consideration of the plant and peculiar rust upon the *solidago.* The plant is widely distributed. It is comparatively harmless. In fact is useful. It is described as being stimulating, diaphoretic, carminative, etc. Some drink the tea and esteem it. But it is not officinally recommended as a remedy. Horses do not like it.

But why did these horses crave it? It was craved for the rust's sake; an appetite had been acquired for it. The taste once accidentally experienced, the animal easily acquires the habit of eating it. It seems to have been brought about in this way: The Summer of 1891 was peculiar, in that there were, especially in highland regions, a succession of showers followed by hot sunshine. This condition peculiarly favored the development of all kinds of vegetable fungus. As a result of this, there was an abundance of rust upon the solidago. In the fields mentioned there was an unusual amount of the golden rod. It was fallow ground and bore more than the usual quantity found in other fields.

The rust spores fell from the solidago upon the succulent grasses underneath. It was easily taken into the system with these grasses. Once in the system it excites a morbid appetite. The afflicted animals recognized and sought for it. The more the system came under its influence the more was it craven; after the manner of the habitual drunkard.

The rust is a vegetable fungus. It is the *Coleosporium Solidaginis*, Thumen (Scheinmitz). The fungus consists of colonies of microscopic vegetable parasites. These, to be appreciated, must be studied under a powerful microscope.

Of its propensities nothing is known. There has been no chance to know anything; no experiments having, to our knowledge, been tried. It has been thus far in the hands of scientific men who pay attention, among other things, to the naming and classification of nature's appearances in rust and fungus. But it is a great work. It prepares the way for the discovery of the nature and powers of these peculiar products.

We little dream of the possible virtues of the vegetable fungus—thousands of species that have only been named, but of whose properties nothing is known. If we reason by analogy, from the few we know, it is not far to see that these products may revolutionize present remedies and take their place. Among the few we know is ergot. It is an example of the power of that class of remedies.

But while there is nothing known of the poisonous properties of the *Coleosporium*, while it is only one of thousands of which we are ignorant, yet there is nothing to show that it is not a poison. There is simply nothing on record. If it can be conclusively shown that the cases herein described were due to other causes, then it does not prove that the *Coleosporium is not a poison* or that it may not have a powerful effect upon the animal economy. But if it cannot be shown that the symptoms occurred otherwise, or if it can be demonstrated by experiments that the fungus taken into the system will produce the same or similar symptoms, then we have had under consideration a hitherto unknown poison.

It is for this purpose I present this paper to you to-day. To elicit discussion and obtain information and experience that will either cause the idea to become established or be displaced by better knowledge.

But closer examination of better specimens of the rust than at first examined, revealed that the *Coleosporium* was itself infested by a second parasite.

The *Dlarluca filum*, castagne (iv.) This parasite belongs to a more poisonous company, but of its actions or properties nothing is positively known. It is open for investigation. It is known to be a poison, but not what kind of a poison. But the idea of its being a poison being a fact, and it infesting the *Coleosporium*, strengthened my idea that I was dealing with the mischief-maker.

Accordingly I began some experiments. I could not (at the late hour I undertook it) collect enough upon the specimens with which to feed an animal. I could not get an animal in time, and by the time I had secured one the frost had killed some of the specimens. We then collected some fine specimens and carefully scraped off the rust. This was placed in a common porcelain mortar and rubbed up in water. It made a reddish, yellow-looking fluid. This was carefully sealed and set away.

Our first experiment was upon a dog. The hypodermic syringe was first cleansed in boiling water. Then 40 drops were injected under the skin. In one quarter hour the little fellow begun to show signs of intoxication. He could not walk without falling over, so he chose to sit or lie quiet. These symptoms lasted about two hours, then passed away, but not without leaving the animal more dull and weak.

No symptoms of pain were noticed. The appetite was not impaired, and the next day the dog appeared all right. As I did not wish to destroy this little pet it was excused from any more experiments. I was convinced that I was dealing with a deadly poison.

We next secured some large rabbits. The first one was given two drachms of the fluid. It died in five hours. It was thoroughly dazed until dead. It showed on post-mortem a pale and flabby appearance of the internal organs. That the blood corpuscles had been interfered with, and that a profound anæmia was being produced, could not be doubted.

Other cases were not injected so freely; 10 drops were administered 3 times daily. To one 10 drops were given once daily. The animals all died, but in varying intervals of time. The appetite in each case remained good until toward the end. But rapid emaciation was apparent in each case, the animals surviving but from 3 to 5 days only. There was a steady decline of the vital powers, and the animals were still alive after they were too weak to move about.

In each case a post-mortem examination was made and the symptoms seen in the horses were verified. There were but insignificant difference in the gross appearances. In no case were there any symptoms of pyæmia or septicæmia. These experiments were made carefully upon six rabbits and one dog.

It is admitted that the method of experiment of hypodermic injection is open to criticism. We employed it as a test merely because we could not test it in any other way. The experiments were simple, but were very carefully made. They yielded results which, if it can be said do not positively prove the poisonous character of the *Coleosporium Solidaginis*, yet they no doubt materially strengthen the position first assumed in this paper. It remains and it shall be done this season to collect enough specimens and feed them directly to a horse. This method will exclude any possible chance from error.

In regard to the treatment of such cases it is impossible to prescribe intelligently. When we do not know a poison sufficiently, we do not know how to combat it. We can only simply follow the indication of nature and build up the system. The vegetable fungus probably enters the blood and, as a proximate principle, becomes a part of the same. Hence it is difficult to attach it. In the cases outlined here it appears to have fed upon the blood corpuscles faster than they could be restored by nature and a vigorous appetite. Probably there was also influence exerted to prevent the formation of new corpuscles. At any rate the blood was very rapidly consumed.

I am indebted to Dr. H. H. Rusby, of New York City, for information regarding the name of the fungus and its known history. Also for the encouragement given me by that learned medical botanist that the simple fungus before us was probably the cause of the mischief described.

SCRANTON, Pa., March 5, '93,
311 Spruce Street.

